# Psychosocial problems caused by abdominal aortic aneurysm surveillance: A cross-sectional survey

**DOI:** 10.1177/09691413251333967

**Published:** 2025-04-15

**Authors:** Jane Hughes, Elizabeth Lumley, Alan Elstone, Jo Hall, Jonathan Michaels, Akhtar Nasim, Steve Radley, Phil Shackley, Niall MacGregor Smith, Gerry Stansby, Emily Wood, Alicia O’Cathain

**Affiliations:** 1 SCHARR, 152607 The University of Sheffield, Sheffield, UK; 2 6634 University Hospitals Plymouth NHS Trust, Plymouth, UK; 3 232173 Derbyshire Community Health Services NHS Foundation Trust, Chesterfield, UK; 4 Sheffield Vascular Institute, Northern General Hospital, Sheffield, UK; 5 c/o PCAAAS, 575008 The University of Sheffield, Sheffield, UK; 6 5983 University of Newcastle and Newcastle upon Tyne Hospitals NHS Foundation Trust, Newcastle Upon Tyne, UK

**Keywords:** Abdominal aortic aneurysm, psychosocial, quality of life, surveillance

## Abstract

**Objective:**

People with abdominal aortic aneurysms (AAA) are at risk of aneurysm rupture, which is immediately life-threatening. People diagnosed with AAA that are a sub-threshold size for intervention undergo regular ultrasound surveillance in England. However, surveillance may cause psychosocial problems such as anxiety. We aimed to use an AAA-specific measure of quality of life to identify the characteristics of people in surveillance with AAA-related psychosocial problems.

**Setting:**

In the National Health Service (NHS) in England, all men are screened for AAA aged 65. They undergo annual surveillance if a small AAA is detected (3–4.4 cm) and three-monthly surveillance if a medium AAA is detected (4.5–5.4 cm). Men with larger AAAs are referred to vascular services.

**Methods:**

A postal survey of men in AAA surveillance from five regional screening centres was conducted using the e-PAQ-AAA quality of life measure which included the Psychological Consequences of Screening Questionnaire.

**Results:**

The response rate was 64% (734/1156). The majority of men reported no AAA-related anxiety or impact on daily living, and no screening-related psychological consequences. However, 11% (29/257) of men in three-monthly surveillance reported having AAA-related anxiety most or all of the time. Men with higher levels of anxiety and physical, emotional or social consequences of surveillance tended to be younger, from more socially deprived communities, have poorer physical health, and have relatively larger and faster-growing AAAs.

**Conclusions:**

Psychosocial problems related to AAA surveillance were not common but did affect a minority of men significantly. An intervention would be beneficial in helping men in AAA surveillance to manage such problems.

## Introduction

Around 4% of men aged between 65 and 74 in England have an abdominal aortic aneurysm (AAA) and this results in around 3000 deaths per year.^1^ Screening can detect the presence of an unruptured AAA allowing elective repair. Screening has been shown to reduce AAA-related mortality and is cost-effective.^2^

Several countries have introduced screening programmes for AAA. Sweden and the four nations in the United Kingdom have national screening programmes provided by the National Health Service (NHS). The NHS AAA Screening Programme in England is offered only to men because women have a much lower incidence of AAA.^2^ Men found to have a large AAA (5.5 cm or greater) are referred to a vascular centre for assessment and surgical consideration. Those found to have a small (3.0–4.4 cm) or medium aneurysm (4.5–5.4 cm) undergo annual or 3 monthly surveillance, respectively.

The psychosocial consequences of screening are often measured for different screening programmes to assess whether harms outweigh benefits.^3^ The evidence for AAA screening and surveillance is mixed. A systematic review of the psychological consequences of screening-detected AAA found moderate levels of anxiety.^4^ Qualitative studies have found that men suffered shock, anxiety and uncertainty,^5–8^ even though they were glad they were in surveillance.^8^ Later qualitative studies have also found heightened anxiety and described how it impacts on men's health-related quality of life^9^ as well as their partners.^10^ A narrative review identified inferior quality of life among men with screen-detected AAA, compared to those without the diagnosis and the general population.^11^ Conversely, a recent systematic review, with meta-analysis, concluded that current evidence does not support a negative impact on health-related quality of life from being in AAA surveillance.^12^

The contradictory evidence base may be due partly to the use of generic quality of life measures which may not be sensitive to screening-related harms.^13^ AAA-specific quality of life measures have been developed^9,14^ and may identify more negative consequences of screening for men with AAA compared with men with normal screens.^15^ Studies have not determined the characteristics of men with higher levels of negative consequences of screening. Therefore, the aim of this study was to identify the characteristics of men with AAA-related psychosocial problems using a AAA-specific measure of quality of life.

## Methods

### Design

A cross-sectional postal survey was conducted. This was undertaken as part of a wider research study ‘Improving the quality of care for men with AAA, who undergo regular screening: reducing the psychosocial consequences of screening and developing a patient-centred exit strategy from surveillance’ (Patient-centred AAA study: PCAAAS). Other components, not reported here, include qualitative interviews with men about interventions to manage AAA-related anxiety.

### Approvals

Ethics approval was obtained (Wales REC 6 ref 23/WA/0019) and permission to send questionnaires to men in the national screening programme was obtained from the NHS England Screening Research, Innovation and Development Advisory Committee.

### Sampling strategy

We aimed to include men currently in the AAA surveillance programme in England. Screening is offered by 38 regional providers. We approached Clinical Leads at seven regional providers, aiming for geographical and socio-economic diversity to ensure a diverse sample of men. Five providers agreed to participate.

As the aim was to identify the characteristics of men with AAA-related psychosocial problems, rather than the prevalence of problems, we did not request a random sample of men in surveillance. Instead, we used a stratified sampling strategy, aiming to over-sample two groups of men in surveillance who were potentially most likely to be anxious. We sampled all men in three-monthly surveillance in the five regional providers because they had medium sized AAAs (4.5–5.4 cm) that may lead to more anxiety than small AAAs (3–4.4 cm). We also sampled all men who had joined surveillance in the previous year because research has shown that anxiety is present in the first year of surveillance and then reduces to normal levels over later years.^16^ Finally, we requested a random sample of a third group of men who had been in annual surveillance for over a year. We aimed for a sample size of 1200, with an expected response rate of 50%–60%.

### Recruitment and informed consent

In the NHS AAA screening programme, men are asked to give permission to be approached for participation in research when they first enter the programme. The vast majority of men give consent. The five regional screening providers gave the research team a list of names and addresses (including postcodes) of men in the three groups outlined above, who had agreed to be approached for research. The group each man belonged to was not given to the research team because we did not have permission to access this information. Names and addresses were sent to the research team using a secure, encrypted, password-protected data transfer process.

### The questionnaire

We used the electronic Personal Assessment Questionnaire e-PAQ-AAA to measure the psychological consequences of AAA.^14^ This was developed and validated in English and is simple and short. It was developed based on evidence reviews, qualitative interviews and consensus, and underwent thorough psychometric testing.^17^ The e-PAQ-AAA consists of: two AAA-specific domains measuring AAA-related anxiety (AAA-ANX) and impact of AAA on activities of daily living (AAA-ADL); the EQ5D-5L for generic quality of life; and the later addition of the Psychological Consequences of Screening Questionnaire (PCSQ) with three domains measuring the physical, social and emotional impact of attending for screening^18^ which was identified as suitable for AAA screening.^19^

The AAA-ANX domain consists of six items about worry related to the AAA, with a response set of “never” to ‘all of the time’. The AAA-ADL consists of six items, e.g. affects ability to undertake personal roles and responsibilities, with a response set from ‘not at all’ to ‘a lot’. The PCSQ physical domain consists of four items, e.g. trouble sleeping. The PSCQ social domain consists of three items, e.g. withdrawing from those close to them. The PSCQ emotional domain consists of four items, e.g. feeling unhappy or depressed. The version of the e-PAQ-AAA we used did not include an item from the PSCQ emotional domain regarding worry for the future.

In addition, we asked about age, ethnicity, their AAA size, whether their AAA was increasing in size, and how long they had been in the screening programme. At each screening visit, men are given a record card with the size of their AAA and its growth, so we were confident that this information was available to them. At the end of the questionnaire, men were invited to offer any other comments in a free-text section.

We worked closely with our Patient and Public Involvement group of five men to ensure that the questionnaire was set out in an easy-to-read way and that the covering letter was inviting.

### Data collection

The e-PAQ-AAA is an electronic questionnaire used within the wider e-PAQ survey system.^20^ e-PAQ is an interactive, web-based system for use in routine clinical care. We created a paper version to promote a higher response rate in this older population. The research team posted a covering letter, the questionnaire and a reply-paid envelope to each man. The covering letter included a web link for completion online through Qualtrics as an alternative option.^21^ We offered information signposting men to support services if they felt that they needed help. We sent a single reminder to non-responders after three weeks. Data collection took place between September and December 2023.

### Analysis

We were able to test one characteristic of non-response bias. We calculated Index of Multiple Deprivation (IMD) scores from postcodes using the Ministry of Housing, Communities & Local Government English indices of deprivation 2019 tool.^22^ We identified men's quintile and decile of social deprivation. We compared responders with non-responders by IMD quintile and IMD decile using the chi-squared test.

We entered the data from the questionnaires into SPSS Statistics (IBM Corp., V29.0, 2023). We followed instructions by the authors of the measures to calculate scores for the two AAA-specific domains, the three PCSQ domains, and the EQ5D-5L. We compared scores for each of the two AAA-specific domains and the three PCSQ domains by the characteristics of men, their AAA and their screening journey using t-test or ANOVA. We used EQ5D-5L as a characteristic of men because it is a measure of general health; specifically, we used the mobility domain as an indicator of physical health. Then we undertook general linear modelling on each of the two AAA-specific domains and the three PCSQ domains to identify characteristics that independently predicted psychosocial consequences. We used forward stepwise regression until further variables had p-values greater than 0.05. The dependent variables were not normally distributed; using the log of each dependent variable gave the same results. There was no evidence of multicollinearity in the independent variables, with all variance inflation factors less than five. We used R squared to calculate the amount of variation in domain scores explained by the characteristics of men. We also undertook content analysis on the free-text comments to identify themes.

## Results

### Response rate

We were provided with 1161 names and addresses. When we sent out the questionnaires, we were notified that five men were either deceased or too ill to complete their questionnaire. The response rate was 64% (734/1156), with 15 of these completed electronically. Response rates for the five regional providers varied between 56% and 72%.

### Non-response bias

The response rate varied by IMD quintile (*p* = 0.021), with the most deprived quintile having the lowest rate of 54% (124/229) and the least deprived/most affluent having the highest response rate of 67% (132/196). We considered IMD deciles too because they are used by the national screening service in their annual reports. The three most deprived deciles had the lowest response rates of 53%‒56% and the other deciles varied between 60% and 73%.

### Characteristics of the sample

Although the response rate was lower for men from the most socially deprived areas, there was good representation from them in the sample (see Table 1). Only 2% of responders reported that they were from an ethnic minority group. 10% reported severe problems with mobility or were unable to walk. Sixty-two percent of men reported a small AAA (3.0‒4.4 cm) and 38% a medium AAA (4.5‒5.4 cm).

**Table 1.** Characteristics of sample.

Characteristic	Categories	*N* (%)
Age group	65–69	175 (24)
	70–74	295 (41)
	75+	250 (35)
Ethnic group (identified by men in the questionnaire)	White	702 (98)
	Minority ethnic group	11 (2)
IMD quintiles	IMD 1 most deprived	124 (17)
	IMD 2	160 (22)
	IMD 3	159 (22)
	IMD 4	153 (21)
	IMD 5 least deprived/most affluent	132 (18)
Mobility based on EQ5DL	No problems walking	356 (50)
	Slight problems walking	148 (20)
	Moderate problems walking	140 (20)
	Severe problems walking	69 (9)
	Unable to walk	5 (1)
General health		72.9 (20.2)^a^
Size of AAA^b^	3.0–4.0 cm	372 (51)
	4.1–4.4 cm	77 (11)
	4.5–4.9 cm	141 (20)
	5.0+ cm	130 (18)
Perceived rate of growth of AAA	Not changing	176 (24)
	Growing slowly	420 (57)
	Growing quickly	29 (4)
	Not applicable because only screened once	109 (15)
Surveillance frequency	Every three months	266 (37)
	Annually	458 (63)
Time in surveillance programme	Less than one year	94 (13)
	1–2 years	53 (7)
	3–4 years	163 (23)
	5–9 years	323 (45)
	Over 10 years	83 (12)
Study sample groups	Three-monthly surveillance	258 (36)
	First year of annual surveillance	89 (12)
	In annual surveillance over a year	371 (52)

^a^ General health is reported as mean VAS score (SD); VAS = visual analogue scale.

^b^ One hundred and twenty-two men did not know the size of their AAA so where possible we used the screening interval they reported to place them in an appropriate AAA size category.

### Prevalence of psychosocial problems

We did not draw a random sample so cannot report the overall prevalence of psychosocial problems for men in surveillance. Instead, we report the prevalence within each of the three samples in our study.

The AAA-ANX, AAA-ADL and PCSQ domain scores are reported later in the paper. These scores do not communicate the percentage of men reporting psychosocial problems. Therefore, in addition to scoring the measures using their official scoring instructions, we used a project-specific approach to scoring AAA-ANX and AAA-ADL by identifying the percentage of men who mainly selected 0, mainly selected 1, mainly selected 2, or mainly selected 3 for the items in each domain (see Table 2). A majority of men predominantly selected ‘never’ for anxiety caused by AAA (varying between 69% and 89% for the three samples) and ‘not at all’ for the effect of AAA on activities of daily living (varying between 85% and 95% for the three study samples). The percentage of men reporting problems was higher in the three-monthly surveillance sample than the other two samples (no statistical test was undertaken because we have not used the official scoring system for measures). This analysis indicates that psychosocial problems were not common but did affect a minority of men most or all of the time. In particular, approximately one in 10 men in three-monthly surveillance were anxious most or all of the time. Reported effects on activities of daily living were less common; rates appeared to be higher in the three-monthly surveillance sample, with one in 20 men reporting being affected ‘moderately’ or ‘a lot’.

**Table 2.** Percentage of men reporting effects on anxiety and activities of daily living for the three study samples.

Domain	Response set	Three-monthly surveillance	First year of annual surveillance	Annual surveillance for over 1 year
		*N* (%)	*N* (%)	*N* (%)
AAA-Anxiety	0 = never	177 (69)	69 (79)	329 (89)
	1 = occasionally	51 (20)	11 (13)	32 (9)
	2 = most of time	26 (10)	7 (8)	9 (2)
	3 = all of the time	3 (1)	0 (0)	1 (<1)
AAA-ADL	0 = not at all	210 (85)	81 (92)	347 (95)
	1 = a little	26 (11)	6 (7)	10 (3)
	2 = moderately	10 (4)	1 (1)	6 (2)
	3 = a lot	1 (<1)	0 (0)	0 (0)

E-PAQ-AAA scores for each domain are reported and compared for each study sample (see Table 3). Higher scores indicate that men reported more problems. The-three monthly sample (men with a medium-sized AAA attending for a scan every three months) had statistically significantly higher scores for all domains than the other two samples (men in their first year of surveillance and men in surveillance for over a year).

**Table 3.** e-PAQ-AAA scores for each domain for the three study samples.

Domain	Three monthly surveillance	First year of annual surveillance	Annual surveillance for over a year	*p*
	Score (*N*)	Score (*N*)	Score (*N*)	
AAA-ANX	25.86 (256)	19.81 (87)	15.31 (371)	< 0.001
AAA-ADL	11.81 (247)	6.94 (88)	4.65 (362)	< 0.001
PCSQ-Emotional	0.64 (247)	0.51 (88)	0.34 (363)	< 0.001
PCSQ-Social	0.50 (248)	0.37 (88)	0.26 (363)	< 0.001
PCSQ-Physical	0.57 (248)	0.36 (88)	0.38 (363)	0.002

### Psychosocial problems by men's characteristics

We compared scores for each domain by the characteristics of men (see Table 4). For all domains, psychosocial problems were more likely to be reported by younger men (65–69 years old), men from socially deprived communities, and men with moderate or severe mobility problems (as a proxy for physical health). Scores were higher for men from ethnic minority communities, but statistical power was low due to small numbers of men from ethnic minority groups in the sample, so this comparison was not statistically significant.

**Table 4.** Scores for psychosocial problems by men's characteristics.

Characteristic	AAA-ANX	AAA-ADL	PCSQ-Emotional	PCSQ-Social	PCSQ-Physical
	Score (*N*)	Score (*N*)	Score (*N*)	Score (*N*)	Score (*N*)
Age group					
65–69	23.3 (173)	9.1 (171)	0.44 (171)	0.36 (171)	0.33 (171)
70–74	19.1 (293)	7.9 (287)	0.34 (288)	0.24 (288)	0.30 (288)
75+	17.8 (250)	5.5 (242)	0.26 (242)	0.22 (243)	0.25 (244)
*p-value*	0.002	0.031	0.016	0.024	0.205
Ethnicity					
White	19.5 (700)	7.4 (682)	0.33 (683)	0.26 (684)	0.28 (685)
Ethnic minority	21.0 (13)	8.9 (15)	0.42 (15)	0.47 (15)	0.41 (15)
*p-value*	0.762	0.692	0.615	0.160	0.357
IMD quintile					
1 (Most deprived)	25.6 (124)	14.1 (122)	0.59 (122)	0.50 (122)	0.53 (122)
2	19.1 (158)	7.2 (154)	0.36 (154)	0.25 (155)	0.29 (155)
3	18.3 (157)	6.8 (153)	0.30 (155)	0.22 (155)	0.26 (155)
4	17.8 (153)	5.0 (148)	0.25 (148)	0.23 (148)	0.22 (148)
5 (Most affluent)	17.3 (131)	4.9 (129)	0.21 (128)	0.14 (128)	0.17 (129)
*p-value*	0.001	< 0.001	<0.001	<0.001	<0.001
Mobility					
No problems	17.0 (353)	3.5 (351)	0.20 (351)	0.14 (351)	0.15 (352)
Slight problems	19.9 (148)	7.5 (144)	0.37 (144)	0.27 (145)	0.30 (145)
Moderate	24.1 (139)	13.4 (133)	0.54 (133)	0.44 (133)	0.50 (133)
Severe	25.4 (69)	16.0 (68)	0.56 (69)	0.52 (69)	0.56 (69)
Unable to walk	11.0 (5)	15.4 (5)	0.35 (5)	0.40 (5)	0.45 (5)
*p-value*	< 0.001	< 0.001	< 0.001	< 0.001	< 0.001

### Psychosocial problems by characteristics of AAA and surveillance experience

We compared scores for each domain by characteristics of the AAA and men's surveillance experience (see Table 5). For all domains, psychosocial problems were more likely to be reported by men with larger aneurysms, men with growing aneurysms, especially faster growing, and men in three-monthly surveillance. Although another study had shown that men in the first year of surveillance had lower quality of life than the general population, in our study sample there was little difference between men in the first year of surveillance compared with longer surveillance experience.

**Table 5.** Prevalence of psychosocial problems by AAA and surveillance characteristics.

Characteristic		AAA- ANX	AAA-ADL	PCSQ Emotional	PCSQ Social	PCSQ Physical
		Mean (*N*)	Mean (*N*)	Mean (*N*)	Mean (*N*)	Mean (*N*)
Size of AAA	3.0–4.0 cm	15.1 (375)	4.1 (370)	0.25 (370)	0.18 (370)	0.21 (371)
	4.1–4.4 cm	18.6 (77)	8.2 (75)	0.30 (76)	0.24 (76)	0.31 (76)
	4.5–4.9 cm	24.0 (140)	10.9 (134)	0.49 (134)	0.40 (134)	0.38 (134)
	5.0+ cm	28.1 (129)	13.1 (125)	0.46 (125)	0.37 (126)	0.40 (126)
	*p-value*	< 0.001	< 0.001	< 0.001	< 0.001	0.001
Growth rate	Not changing	13.1 (175)	3.3 (170)	0.20 (169)	0.16 (169)	0.17 (170)
	Growing slowly	20.5 (419)	8.0 (410)	0.36 (412)	0.28 (413)	0.31 (413)
	Quickly	40.7 (28)	23.6 (27)	0.72 (27)	0.64 (27)	0.66 (27)
	Do not know	19.9 (63)	7.4 (62)	0.33 (62)	0.27 (62)	0.28 (62)
	*p-value*	<0.001	<0.001	< 0.001	<0.001	<0.001
Surveillance frequency	Every 3 months	26.0 264	11.9 (255)	0.48 (255)	0.39 (256)	0.38 (256)
	Annual	15.8 456	4.9 (448)	0.26 (449)	0.19 (449)	0.24 (450)
	*p-value*	<0.001	<0.001	<0.001	<0.001	0.001
Number of years in annual or three-monthly surveillance^a^	<1 year	19.7 (92)	6.9 (93)	0.39 (93)	0.31 (93)	0.24 (93)
	>1 year	19.6 (620)	7.5 (602)	0.33 (603)	0.26 (604)	0.30 (605)
	*p-value*	0.944	0.694	0.439	0.433	0.374

^a^ Note that this variable was collapsed into two categories because of the existing evidence base about first year versus other years.

### Characteristics independently associated with psychosocial problems

We tested for independent contribution of characteristics for each domain of psychosocial problems and had fairly similar findings for each domain: that men with poor mobility (as a proxy for poor physical health), men from deprived communities, younger men, and men with larger aneurysms were more likely to report psychosocial problems (see Online Appendix). The rate of growth of AAA also independently explained variation in AAA-ANX and AAA-ADL. The amount of variation explained by variables was small, with R-squared varying between 14% and 23%.

### Free-text comments

Men completing the questionnaire had the option of completing a free-text section on any aspect of having a AAA or being in surveillance. A total of 34% (246/734) completed this section, usually writing a sentence or two. We identified four themes: impact of comorbidities, AAA-related anxiety, impact of being in surveillance for AAA, and information provision.

#### Impact of comorbidities

Comorbidities such as cancer, arthritis and cognitive issues were reported by 34% (84/246) of men as having more impact on them than their AAA diagnosis. They stated that their AAA was not a cause for worry or anxiety for them, relative to these other conditions. A few men reported that having a diagnosis of AAA was an additional worry on top of their other comorbidities. Some also commented that when completing the questionnaire, particularly the questions directly related to their feelings about having a diagnosis of AAA and being in surveillance, it was difficult to separate the impact of having an AAA from the impact of their other comorbidities, which may have affected the boxes they ticked.

I have numerous health & physical conditions. They are way more extensive than an aneurysm. [Small AAA, annual screening]

#### AAA-related anxiety

In relation to anxiety, 20% (50/246) of men commented on how being diagnosed with AAA and being in surveillance made them feel. Many said they were not worried or anxious at all. For some men, it was a shock being told they had a AAA, and they described it as having a significant impact on their lives. For those with experience of a family member or friend who had died from a ruptured AAA, being diagnosed themselves with a AAA made them more aware of, and more anxious about, the potentially negative outcomes.

I have had no problems but was in shock when I found out I had this AAA. Although small I will definitely keep my routine appointments in case it starts to grow. [Small AAA, annual screening]

[the aneurysm] is now getting very close to needing surgery, so obviously I am now a bit more worried than what I was previously. Up until this time I had no worries or problems as I was being checked regularly. [Medium AAA, three-monthly screening]

the very thought that if it goes then it's most likely ‘game over’ … does affect daily life to the extent I have now given up my job of 20 years … solely because of the fear from the physical exertion causing an issue. [Medium AAA, three-monthly screening]

#### Impact of being in surveillance for AAA

In total, 21% (51/246) of comments were related to being in surveillance. Most of these comments described how undergoing scans to measure AAA size, particularly around the time of the next scan appointment, was reassuring. Although some men expressed anxiety, the majority of comments about being in surveillance were positive, both about having the opportunity to be scanned regularly as well as the excellent service provided by NHS screening staff.

I am grateful that the screening service was offered to me. I had no idea I had an aneurysm and, as the size was slowly increasing, I have changed my lifestyle to be fitter and lose weight. I realise that it may grow in the future but feel I am fitter now and certainly slimmer and would be better prepared if intervention was required. [Medium AAA, three-monthly screening]

#### Information provision

Provision of information was referred to by 11% (28/246) of men who commented. This was both in terms of diagnosis and surveillance. Some men did not understand why they could not be operated on whilst their AAA was small, suggesting they did not have a clear understanding of the relative risks of the AAA repair procedure in relation to their aneurysm size. Men reported not knowing what exercise to do, what to avoid, and what symptoms to look out for if the aneurysm was leaking or had burst. Some men stated that they had received sufficient information to meet their needs, and this enabled them to make changes to their lifestyles with the aim of slowing down growth of their aneurysm. Others more generally commented on wanting more information, particularly in order to understand what might happen in the future.

It is not very clear why I would not have the aneurysm operated on, especially as the older I become the more problematic an operation would become. [Small AAA, annual screening]

I can find no specific guidance on the type of exercise to do or which to avoid. [Small AAA, annual screening]

I would like more information about what's going to happen in the future or moving forward? Regarding treatments for AAA, chances of survival of treatments, problems caused to treatments by current health or previous conditions or ongoing conditions. [Medium AAA, three-monthly screening]

## Discussion

### Summary of results

The majority of men in this sample did not report experiencing AAA-related anxiety, effects on activities of daily living caused by AAA, or psychological consequences of attending for AAA screening. However, a minority of men reported significant problems: 11% (29/257) of men in three-monthly surveillance (AAA size 4.5–5.4 cm) reported suffering AAA-related anxiety most or all of the time, and 4% (11/247) reported moderate or a lot of impact on activities of daily living. Men with higher levels of anxiety, diminished activities of daily living, and physical, emotional or social consequences of screening were: younger (aged 65–69), from socially deprived communities, had moderate or severe mobility problems (as a proxy for physical health), and had larger AAAs. Having a fast-growing AAA also increased anxiety. The free-text comments supported these findings and highlighted the need for men to have further information to address psychosocial problems.

### Context of other research

Our findings overall align with those of a recent systematic review showing that current evidence did not support a negative impact on quality of life.^12^ The majority of men in our survey reported little or no impact on quality of life of being in AAA surveillance. We did however identify that a minority of men suffered psychosocial problems caused by AAA screening or surveillance all or most of the time, in line with other research.^4,8^ The key contribution of our research is to move the debate from whether screening and surveillance causes psychosocial problems to identifying the characteristics of the minority of men suffering from AAA-related psychosocial problems.

### Strengths and limitations

The strength of our study was that the survey used a validated AAA-specific instrument rather than a generic quality of life one, and the response rate was good. However, there were limitations. First, we found non-response bias, which is not unusual. The response rate was lower for men from socially deprived communities. Given that men from socially deprived communities reported higher levels of psychosocial problems, the levels reported here are likely to slightly underestimate levels in the population of men in surveillance. A sensitivity analysis for men in three-monthly surveillance showed that if the response rate had been the same in the most deprived quintile as the most affluent quintile (67% rather than 54%) then 12% of men would have reported being anxious most or all of the time rather than 11%. Second, the version of e-PAQ-AAA we used did not include one item from the PCSQ so comparisons with other studies using PCSQ should not be made for the emotional domain of this tool.

### Implications

The evidence base about whether men in AAA surveillance experience higher levels of anxiety or worse quality of life caused by being screened for AAA is mixed. Our study shows that most men do not report problems, but that a minority report significant problems. We recommend the development of an intervention to help these men manage their anxiety. One way forward may be to use the characteristics of men with heightened anxiety reported in this paper to target men who most need an intervention. Alternatively, the intervention could be accessible to all men in surveillance, given that the characteristics we identified explained only a small amount of the heightened anxiety in this sample. Potential interventions might include cognitive behavioural therapy for men with high levels of anxiety, peer support programs, or digital health interventions to provide scalable solutions to address anxiety. The free-text comments highlighted the need for men to have further information. It is possible that a lack of information contributes to psychosocial problems. Indeed, recent studies have concluded that information provision might address the worries and concerns of men in AAA surveillance.^5,12^ In another part of our study, we have interviewed men about potential interventions and will report this in a separate paper.

These findings could be used to inform changes to AAA screening guidelines, particularly in terms of the information that is provided. Services providing AAA screening could consider providing additional support to a sub-group of men identified as experiencing more psychosocial consequences. Our next piece of research explores men's views of the types of interventions that might help them, and the results of this can be used to shape future strategies.

## Conclusion

Psychosocial problems related to having a screening-detected AAA were not common but did affect a minority of men significantly. An intervention is indicated to help men to manage psychosocial problems.

## Supplemental Material

sj-pdf-1-msc-10.1177_09691413251333967 - Supplemental material for Psychosocial problems caused by abdominal aortic aneurysm surveillance: A cross-sectional survey Supplemental material, sj-pdf-1-msc-10.1177_09691413251333967 for Psychosocial problems caused by abdominal aortic aneurysm surveillance: A cross-sectional survey by Jane Hughes, Elizabeth Lumley, Alan Elstone, Jo Hall, Jonathan Michaels, Akhtar Nasim, Steve Radley, Phil Shackley, Niall MacGregor Smith, Gerry Stansby, Emily Wood and Alicia O’Cathain in Journal of Medical Screening
