# Supplementary material for: Psychosocial problems caused by abdominal aortic aneurysm surveillance: A cross-sectional survey
Source: J Med Screen. 2025 Apr 15;32(4):190–7. doi: 10.1177/09691413251333967 (PMC12569146; doi:10.1177/09691413251333967)
Supplement: sj-pdf-1-msc-10.1177_09691413251333967 - Supplemental material for Psychosocial problems caused by abdominal aortic aneurysm surveillance: A cross-sectional survey [file sj-pdf-1-msc-10.1177_09691413251333967.pdf]

Appendix: Changes in parameter estimates compared with the reference category for the five psychosocial domains (based on generalised linear model regressions in SPSS)

Independent variable	AAA-ANX	AAA-ADL	PCSQ-Emotional	PCSQ-Social	PCSQ-Physical
Mobility/physical health	p<0.001	P<0.001	p<0.001	P<0.001	p<0.001
No problems walking	-	-	-	-	-
Slight problems walking	2.4	3.7	0.16	0.13	0.11
Moderate problems walking	7.3	9.9	0.36	0.31	0.35
Severe problems walking	8.2	11.4	0.40	0.41	0.39
Unable to walk	-3.8	14.9	0.32	0.41	0.38
Deprivation	P=0.007		p=0.003	P=0.002	p<0.001
1 (Most deprived)	-		-	-	-
2	-3.5		-0.16	-0.19	-0.17
3	-3.4		-0.17	-0.19	-0.19
4	-5.5		-0.24	-0.17	-0.24
5 (Most affluent)	-5.7		-0.28	-0.28	-0.28
Age	p<0.006	p=0.017	p=0.002	p=0.002	
65 - 69	-	-	-	-	
70 - 74	-5.2	-2.5	-0.12	-0.16	
75+	-6.3	-4.5	-0.21	-0.18	

Size AAA	p<0.001	p<0.001		p<0.001	p<0.001
3.0 - 4.0 cm	-	-		-	-
4.1 - 4.4 cm	2.1	3.0		0.06	0.07
4.5 - 4.9 cm	7.9	6.5		0.25	0.19
5.0 + cm	10.7	7.0		0.18	0.19
Rate of growth AAA	p<0.001	p=0.005			
Not changing	-	-			
Growing slowly	3.2	1.7			
Growing quickly	17.5	10.6			
Only screened once	2.3	1.4			
Frequency of scans			p<0.001		
Annual			-		
3 months			0.24		
R-squared	18%	23%	14%	14%	16%

Each parameter indicates the change in the predicted value of the dependent variable for a unit increase in the independent variable, holding other variables constant. Positive values indicate higher levels of psychosocial problems. Negative values indicate lower levels of psychosocial problem.
